# Metastatic Insulinoma Managed with Radiolabeled Somatostatin Analog

**DOI:** 10.1155/2013/252159

**Published:** 2013-12-17

**Authors:** Ricardo Costa, Rubens Costa, Carlos E. Bacchi, Paulo Almeida Filho

**Affiliations:** ^1^Department of Oncology, Real Hospital Portugues, 52010 Recife, PE, Brazil; ^2^Cedar Valley Cancer Center, Waterloo, IA 50701, USA; ^3^Laboratorio Bacchi, 18602 Botucatu, SP, Brazil; ^4^Department of Nuclear Medicine, Real Hospital Portugues, 52010 Recife, PE, Brazil

## Abstract

Insulinoma is a rare pancreatic neuroendocrine tumor. Overproduction of insulin and associated hypoglycemia are hallmark features of this disease. Diagnosis can be made through demonstration of hypoglycemia and elevated plasma levels of insulin or C-Peptide. Metastatic disease can be detected through computerized tomography (CT) scans, magnetic resonance imaging (MRI), and positron emission tomography (PET)/CT. Somatostatin receptor scintigraphy can be used not only to document metastatic disease but also as a predictive marker of the benefit from therapy with radiolabeled somatostatin analog. Unresectable metastatic insulinomas may present as a major therapeutic challenge for the treating physician. When feasible, resection is the mainstay of treatment. Prevention of hypoglycemia is a crucial goal of therapy for unresectable/metastatic tumors. Diazoxide, hydrochlorothiazide, glucagon, and intravenous glucose infusions have been used for glycemic control yielding temporary and inconsistent results. Sandostatin and its long-acting depot forms have occasionally been used in the treatment of Octreoscan-positive insulinomas. Herein, we report a case of metastatic insulinoma with very difficult glycemic control successfully treated with the radiolabeled somatostatin analog lutetium (^177^LU).

## 1. Introduction

Insulinoma is a rare pancreatic neuroendocrine tumor [1]. Overproduction of insulin and associated hypoglycemia are hallmark features of this disease. Diagnosis can be made through demonstration of hypoglycemia and elevated plasma levels of insulin or C-peptide. Confirmation of neuroendocrine nature of the tumor by immunohistochemistry requires chromogranin and synaptophysin positivity. Metastatic disease can be detected through computerized tomography (CT) scans, magnetic resonance imaging (MRI), and positron emission tomography (PET)/CT [2]. Somatostatin receptor scintigraphy may be used not only to document metastatic disease but also as a predictive marker of benefit from therapy with radiolabeled somatostatin analog [3].

The treatment of unresectable metastatic insulinoma is associated with prolonged hospitalization for intravenous glucose infusion. The management of recurrent hypoglycemia also encompasses the administration of diazoxide, hydrochlorothiazide, and glucagon.

Sandostatin and its long-acting depot form (Sandostatin LAR) are an adjunct therapy to neuroendocrine tumors. There are a few reports of glycemic control in patients with insulinoma treated with radio labeled somatostatin analogs [4, 5].

The m-TOR inhibitor everolimus has been used to control hypoglycemia in this group of patients [5–7]. Moreover, it has recently shown efficacy in combination with Sandostatin in the therapy of neuroendocrine tumors [8].

Herein, we report a case of metastatic insulinoma successfully treated with the radiolabeled somatostatin analog lutetium (^177^LU).

## 2. Case

The patient was a 32-year-old otherwise healthy female who was admitted to a tertiary care center with unexplained hypoglycemia and altered mental status. She was in her usual state of health until one month prior to hospitalization when she presented with morning drowsiness and loss of appetite. Due to the gradual worsening of symptoms, she was brought to the Emergency Department for further evaluation.

**Figure 1:**
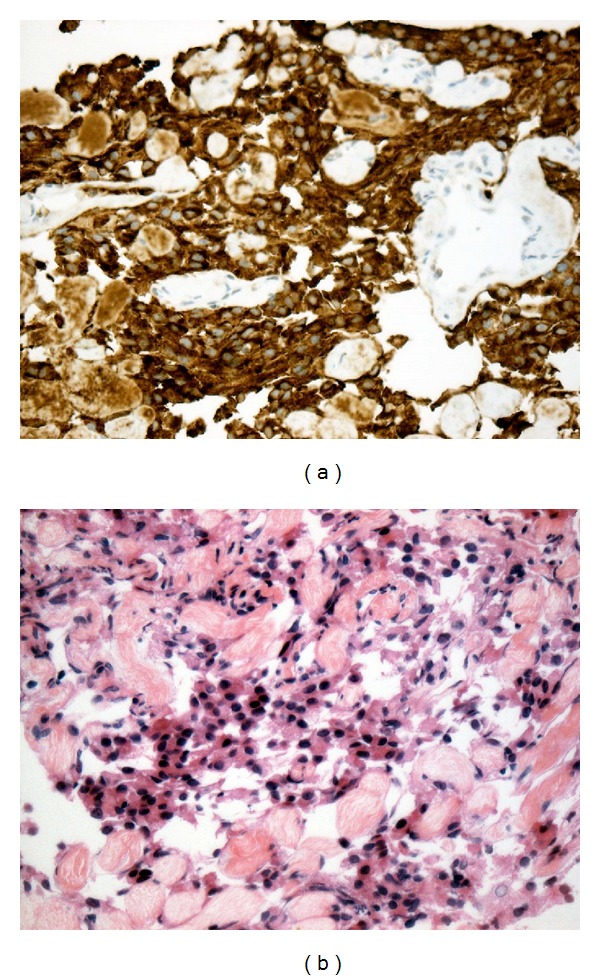
Low-grade neuroendocrine neoplasia of the pancreas. (a) Strong and diffuse expression of chromogranin A. (b) By immunohistochemistry.

Upon admission, the patient was found to have a glucose level of 35 mg/dL. She had recurrent episodes of hypoglycemia despite numerous intravenous glucose infusions which led to a work up for a possible pancreatic tumor. An ultrasound of the abdomen was performed showing several liver nodules along with a large pancreatic mass measuring up to 10.7 cm in the largest diameter (not shown). Computerized tomography (CT) scan of the abdomen was performed confirming the sonographic findings. The patient underwent a liver biopsy. Histopathology and immunohistochemistry results were consistent with low-grade neuroendocrine neoplasia (positive stain for chromogranin A) (Figure 1). Insulin and C-peptide levels are depicted in Table 1.

The patient received continuous intravenous glucose because of recurrent episodes of hypoglycemia throughout the hospital stay (Figure 5). Glucagon and hydrochlorothiazide failed to elevate glucose levels.

**Figure 2:**
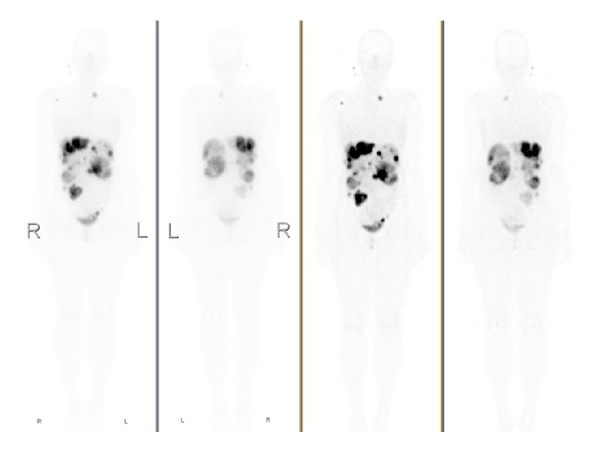
Octreoscan: high uptake in focal areas of the liver, left lower quadrant of the abdomen, and cervicothoracic area.

Octreoscan was performed showing a high uptake in focal areas of the liver, left lower quadrant of the abdomen, and cervicothoracic area (Figure 2).

**Figure 3:**
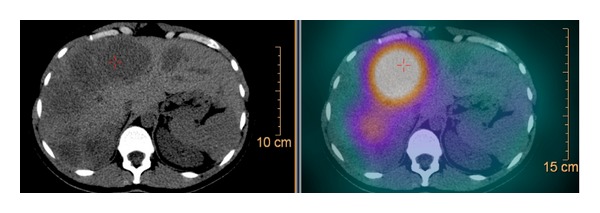
Octreoscan SPECT/CT prior to radiolabeled somatostatin analog lutetium (^177^LU) administration.

**Figure 4:**
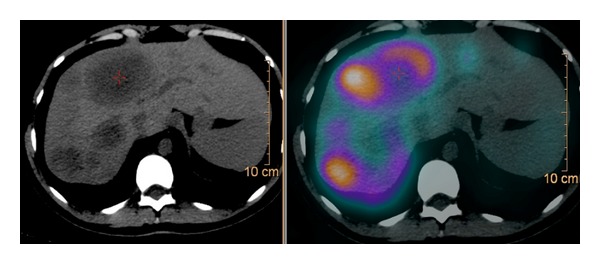
Octreoscan SPECT/CT after 1st infusion of radiolabeled somatostatin analog lutetium (^177^LU) administration.

**Figure 5:**
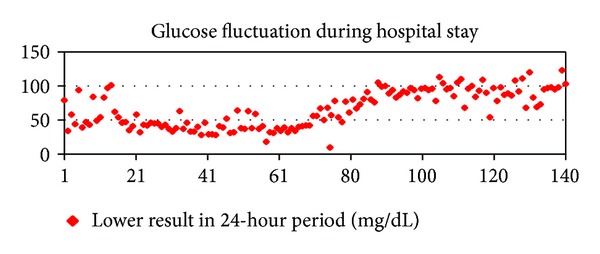


Sandostatin LAR 30 mg IM was started on hospital stay day 43 on an every 28 days basis. The patient received 2 infusions of radiolabeled somatostatin analog lutetium (^177^LU) 8 weeks apart beginning on hospital day 56. By the second administration of the radiopharmaceutical, Octreoscan SPECT/CT had already shown objective metabolic and radiologic response to treatment (Figures 3 and 4). Serum glucose levels started to rise by hospital stay day 60 (Figure 5). Intravenous glucose infusion was titrated progressively down onwards. The patient was discharged on a normocaloric diet on hospital day number 140.

## 3. Discussion

The management of insulinomas is challenging and requires a multidisciplinary approach. When feasible, resection is the mainstay of treatment. Prevention of hypoglycemia is a crucial goal of therapy for unresectable/metastatic tumors. Diazoxide, hydrochlorothiazide, glucagon, and intravenous glucose infusions have been used for glycemic control yielding temporary and inconsistent results.

Insulinomas are generally less octreotide avid than other pancreatic neuroendocrine tumors. Octreoscans may be performed if treatment with octreotide and its radiolabeled analogues is being contemplated. In the absence of somatostatin receptors, they may actually aggravate hypoglycemia. Sandostatin and its long-acting depot forms have been used in the treatment of Octreoscan-positive insulinomas [9, 10].

Radiolabeled somatostatin analog lutetium (^177^LU) has been successfully tested in the treatment of gastroenteropancreatic neuroendocrine tumors [3]. Tumor response and progression-free survival compared favorably to cytotoxic chemotherapy. There are anecdotal reports of successful treatment of metastatic insulinoma with this radiopharmaceutical. For instance, van Schaik et al. treated five patients with metastatic insulinoma achieving disease and glycemic control for an average of twenty seven months [11].

Herein, we report a case of metastatic insulinoma with very difficult glycemic control. Temporizing measures including glucagon, hydrochlorothiazide, and continuous intravenous glucose were unsuccessful in raising glycemic levels.

After results of Octreoscan became available, we decided to use radiolabeled somatostatin analog because of previous reports of glycemic control and objective responses. Glucose levels steadily improved, as shown in Figure 5. Moreover, restaging after only one infusion of radiolabeled somatostatin analog lutetium (^177^LU) did show clear evidence of objective response in line with other reports.

In summary, unresectable metastatic insulinomas may present as a major therapeutic challenge for the treating physician. For Octreoscan-positive tumors, radiolabeled somatostatin analog lutetium (^177^LU) may represent an option for glycemic and systemic disease control, as shown in the case presented here.

## Figures and Tables

**Table 1:** 

Insulin level	C-peptide
73,00 uUI/mL Fasting glucose level < 100 mg/dL (2–20 uUI/mL)	4.17 ng/mL (0.48–5.05 ng/mL)
